# Fat-rich diet promotes microbiome-dependent ATP synthesis in sheep model

**DOI:** 10.1186/s40104-025-01214-9

**Published:** 2025-06-05

**Authors:** Fan Hu, Kefyalew Gebeyew, Zhiwu Wu, Bingrui Chen, Jinzhen Jiao, Zhiliang Tan, Di Tian, Zhixiong He

**Affiliations:** 1 State Key Laboratory of Forage Breeding-by-Design and Utilization, National Engineering Laboratory for Pollution Control and Waste Utilization in Livestock and Poultry Production, and Hunan Provincial Key Laboratory of Animal Nutritional Physiology and Metabolic Process, Institute of Subtropical Agriculture, Chinese Academy of Sciences, Changsha, 410125 Hunan China; 2 College of Advanced Agricultural Sciences, University of Chinese Academy of Sciences, Beijing, 100049 China; 3 State Key Laboratory of Efficient Production of Forest Resources, Beijing Forestry University, Beijing, 100083 China; 4 Yuelushan Laboratory, Changsha, 410125 Hunan China

**Keywords:** Fat-rich diet, Gastrointestinal microbial ATP, Metagenome

## Abstract

**Background:**

The ketogenic diet that forces adenosine triphosphate (ATP) production by beta-oxidation of fatty acids instead of carbohydrate glycolysis, has gained consensus on host metabolism. However, the mechanisms how a ketogenic diet alters gastrointestinal microbiome and its downstream consequences on microbial nutrient availability and energy metabolism remain to be elucidated. Here, we used the sheep model fed with fat-rich diet to evaluate the symbiotic microbiome across three regions of the gastrointestinal tract (rumen, ileum, and colon) to gain a comprehensive understanding of the microbial energy metabolism and microbe-mediated ATP biosynthesis.

**Results:**

Results showed that sheep fed a fat-rich diet had a greater ADG and increased reliance on fat oxidation for fuel utilization. Metagenomics analysis showed the loss of the specialized fiber-degrading bacteria (genus_*Fibrobacter*) in the rumen and enrichment of genera *RUG420* and *Eubacterium*, which are involved in lipid metabolism and bile acid processing, in the ileum. A significant functional shift related to energy metabolism was shared across three regions of the gastrointestinal microbiomes. These shifts were dominated by glycolysis/gluconeogenesis and TCA cycle in the rumen and by fatty acid degradation and bile acid transformation in the ileum, indicating adaptation to nutrient availability and energy acquisition. Notably, the abundance of substrate-level phosphorylation (SLP) enzymes was significantly increased in the rumen, ileum and colon, while the ATP-producing capacity through electron transport phosphorylation (ETP) by family_Bacteroidaceae in rumen and Acutalibacteraceae in ileum of sheep with fat-rich diet.

**Conclusions:**

Altogether, the ATP-related microbiome encoding SLP and ETP in rumen, ileum, and colon contributed 36.95% to the host’s weight variation. Our study is the first one demonstrating the microbial potential in the ATP synthesis under the shift in dietary energy source, providing a new perspective on the energy metabolism and precise human macronutrients nutrition.

**Supplementary Information:**

The online version contains supplementary material available at 10.1186/s40104-025-01214-9.

## Introduction

A ketogenic diet, characterized by high-fat and low-carbohydrate intake, has recently gained widespread attention as a promising dietary therapy for human weight loss, anti-inflammatory effects, and even for suppressing cancer [1–4]. It induces a pronounced shift in metabolic fuel utilization that forces adenosine triphosphate (ATP) production through the beta-oxidation of fatty acids instead of the glycolysis process as the main energy source [5]. Nutrient utilization and energy harvest result from the meticulous amalgamation of metabolic machinery by the microbes and the host across the gastrointestinal tract [6, 7]. Although the ketogenic diet has gained consensus on treatment of host metabolism disorder and illnesses [8, 9], the mechanisms of how a ketogenic diet alters gastrointestinal microbiome and its downstream consequences on microbial nutrient availability and energy metabolism are unknown.

The diverse and enormous commensal microbes in the gastrointestinal tract, which have coevolved with the host, not only complement the metabolic and energy-gathering capabilities but also affect site-specific function and physiology [10–13]. The metabolic processes can adapt to dietary fuel supply by altering fuel selection [14]. There is a prioritisation of energy substrate oxidation: the microbial community is primarily involved in carbohydrate metabolism, which predominantly results in the formation of short-chain fatty acids and accounts for a majority of the host’s energy demand. In conditions of limited carbohydrates, bacteria turn to lipids as an alternative energy source [15, 16]. This adaptation to nutritional shifts provides a direct competitive advantage to the microbial community that can utilize specific substrates, rendering them more capable of proliferating in specific gastrointestinal tract [17]. Given these complex interactions, gaining more insight into the relationship between energy substrates and the host-microbiota metabolism is crucial for developing innovative, microbiome-based dietary strategies and optimizing intestinal health.

ATP, the energy currency generated through carbohydrate and lipid catabolism, is the main energy source for microbial growth, rumen microbial protein synthesis, and endergonic metabolic processes [18, 19]. Microbial ATP production occurs in two major mechanisms: substrate-level phosphorylation (SLP) and electron transport phosphorylation (ETP). Microbial species show varying tolerances to the gastroenteric oxygen, which significantly impact microbial growth and energy production mode under regional effects [20]. Under anaerobic conditions, SLP facilitates ATP synthesis through glycolysis, where the high-energy phosphate groups are directly transferred from the substrate molecule to ADP [21]. In contrast, ETP utilizes the electrochemical potential generated by the respiratory chain to drive efficient ATP synthesis via the ATP synthase, representing a more efficient but rate-limiting step [22, 23]. While the importance of both SLP and ETP in energy metabolism is well established [24], the potential for dietary modulation of microbiota-dependent ATP production in the gastrointestinal tract remains largely unexplored.

Considering difficulties in sampling the gastrointestinal tract of human beings and the fact that sheep (*Ovis aries*) are more comparable to humans with respect to body weight, fetal development, offspring number, and physiology, the sheep model is a research model closer to humans than mice for studying metabolism disorder, cardiovascular disease, and central nervous system [25–27]. In the present study, we used shotgun metagenomics to profile microbiome structure and function for energy productivity from content samples of three gastrointestinal regions in sheep fed carbohydrate-rich versus fat-rich diets. Our study aims to employ metagenomic sequencing to (1) decipher the microbial potential in the ATP synthesis and their taxonomy assignment, (2) compare the ATP-producing capacity of region-specific microbiota, and (3) identify fat-rich diet-induced modifications of gastrointestinal microbiota and ATP productivity. The findings may lay the foundation for understanding the role of energy metabolism in shaping the entire gastrointestinal microbiome, and provide important clues for precise human macronutrients nutrition.

## Materials and methods

### Animals and experimental design

The feeding trial was carried out at the Ecological Grass and Animal Husbandry Engineering Experimental Station, Chinese Academy of Sciences (Hulunbuir City, Inner Mongolia Autonomous Region, China). Twenty Hulunbuir sheep at 3-month-old (BW = 17.71 ± 0.43 kg) were obtained and fed ad libitum for 2 weeks to determine the additive ratio of palm fat powder and ensure the normal and healthy physiological state of the host. After the adaptation period, 20 sheep were assigned into two dietary groups with equal numbers of males and females: one was fed with a normal carbohydrate-rich diet (CR) consisting mainly of 36.95% corn, 14.93% soybean meal, and 4.86% wheat bran; the other received a fat-rich diet (FR) consisting of 16.34% corn, 13.94% soybean meal, 4.54% wheat bran and 23.65% palm oil powder for 8 weeks. Dietary ingredients and nutrient composition were presented in Table S1. The diet was offered at 2% body weight (BW) twice daily, at 8:00 and 16:00 pm, allowing 5%–10% orts, and water was provided ad libitum. Feed intake was monitored daily throughout the experiment. Each morning, the initial weight of feed provided to each animal was recorded. Prior to the next morning’s feeding, the remaining feed in each pen was carefully weighed. Daily feed intake per animal was calculated as the difference between the initial feed weight and the weight of remaining uneaten feed. Each animal’s live weight was measured every 2 weeks before morning feeding to account for differences in gut fill. Total weight gain was computed as the difference between final and initial body weight. The average daily gain was calculated by dividing the total body weight gain by the number of feeding days.

### Sample collection

Blood samples were collected from the jugular vein before morning feeding every two weeks. The samples were centrifuged at 3,000 × *g* for 15 min at 4 °C to collect the serum, separated into three tubes, and then stored at −20 °C for subsequent biochemical index analyses. The serum samples were analyzed using a 7020 auto-analyzer (TBA-120 FR, Toshiba Medical Systems Corporation, Tokyo, Japan) to determine the total protein (TP), blood urea nitrogen (BUN), alanine aminotransferase (ALT), aspartate aminotransferase (AST), alkaline phosphatase (ALP), albumin (ALB), triglyceride (TG), glucose (GLU), cholesterol (CHOL), high-density lipoprotein cholesterol (HDL), low-density lipoprotein cholesterol (LDL). The non-esterified fatty acids (NEFA) in serum were evaluated (Nanjing Jiancheng Bioengineering Institute, Nanjing, China) following the manufacturer’s instructions.

To decrease the effect of sampling time points, each sheep was slaughtered 4 h after morning feeding on the final day of week 8. The gastrointestinal tract (GIT) regions, including rumen, ileum, and colon, were tied off using cotton rope, and then the content samples from each region were collected. Afterward, the collected samples were snap-frozen in liquid nitrogen and then stored at −80 °C for further analysis.

### The apparent digestibility of nutrients

The apparent digestibility was assessed using the entire faecal collection method, consisting of 7 d for data collection. Faecal samples were collected before morning feeding, then 10 mL of 10% (v/v) sulfuric acid was added to the fresh faecal to prevent ammonia nitrogen volatilization. Feed and faeces were oven-dried at 65 °C for 72 h, and smashed using a mill with a 1-mm screen in order to analyze the dry matter (DM), crude protein (CP), ether extract (EE), neutral detergent fiber (NDF), acid detergent fiber (ADF), and crude ash. The gross energy of the feed was measured using a bomb calorimeter (6400, PARR Works Inc., USA). The NDF and ADF were assayed following the method of Van Soest et al. [28].

### DNA extraction, shotgun sequencing, and bioinformatics analysis

Microbial DNA was extracted from rumen, ileum, and colon content samples using the E.Z.N.A.^®^ stool DNA Kit (Omega Bio-tek, Norcross, GA, USA) according to manufacturer’s protocols. High-quality DNA from each sample was then used to construct a metagenomic library with an insert size of 350 bp according to the manufacturer’s instructions for the TruSeq DNA PCR-Free Library Preparation Kit (Illumina, San Diego, CA, USA), and run on the NovaSeq 6000 platform (Illumina).

For the metagenomic analysis, the raw sequences underwent quality control (Trimmomatic, version 0.36), assembly (MEGAHIT, version 1.1.1), prediction (METAProdigal, v2.6.3), and clustering into a non-redundant gene set (BWA-MEM, version 0.7.17). We systematically examined the rumen, ileum, and colon microbiome structure and function using shotgun metagenomic sequencing. From a total of 11.9 billion high-quality reads, the metagenomic data was obtained with 21.9 million contigs and 47.3 million open reading frames (ORFs) via metagenomic assembly and ORF prediction (Table S2). The annotations of the gene set were performed with KEGG and NCBI-NR using DIAMOND (v0.9.22), and abundance profiles of genes were calculated in transcripts per million (TPM), with corrections for variations in gene length and mapped reads per sample.

### The major contributors to SLP and ETP enzymes in the microbiome

There are 3 kinds of SLP enzymes: phosphoglycerate kinase (EC 2.7.2.3), pyruvate kinase (EC 2.7.1.40) in glycolysis, succinyl-CoA synthetase (EC 6.2.1.5) in the TCA cycle, acetate kinase (EC 2.7.2.1), and butyrate kinase (EC 2.7.2.7). The ETP is composed of a series of transmembrane ion pumps and ATPase. We defined these KOs that perform the function of substrate-level phosphorylation (SLP) and electron transport phosphorylation (ETP) as a function role in ATP synthesis. The relative abundances of annotated taxa (phylum and family level) and KOs were calculated according to the gene abundance based on KEGG and NR annotation.

### Statistical analysis

The data on growth performance and the apparent digestibility of nutrients were analyzed using *t*-tests. For analysis of serum biochemistry parameters, the statistical significance of the differences between the two curves was determined by two-way repeated measures ANOVA and a Bonferroni post hoc test. Alpha (richness index) and beta (Bray-Curtis dissimilarity) diversities indices were calculated based on the taxonomic profile (species) using the R vegan package. Bray-Curtis distances between the two dietary groups, based on the taxonomic profiles (species) and function (KO genes), were defined. The differences between two diet groups were assessed using the permutational multivariate analysis of variance (PERMANOVA) in the R vegan package (v.2.5-6) with 9,999 permutations and then visualized using a PCoA plot. The nonparametric Scheirer-Ray-Hare extension of the Kruskal-Wallis test was used to test two independent factors (region and diet) and the influence of their interaction on taxonomic and functional variance. The taxonomic and functional matrices based on KEGG pathways and genes were compared between two diet groups using the Wilcoxon rank-sum test. All *P* values in metagenomic analysis were adjusted for False Discovery Rate (FDR) using the Benjamini-Hochberg method. Statistical significance was declared at *P* < 0.05 and tendencies at 0.05 ≤ *P* ≤ 0.10.

To evaluate the relative importance of ETP-related microbiome and SLP-related microbiome in host phenotype, multiple regression on matrices (MRM) analysis was utilized. The matrices of microbial community were calculated using Bray-Curtis distance. The coefficients of the regression could be used to approximate the degree of the influence of different explanatory variables. MRM was performed using the R package ecodist with 10,000 permutation tests.

## Results

### The fat-rich diet promoted higher growth performance and enhanced lipid metabolism

**Fig. 1:**
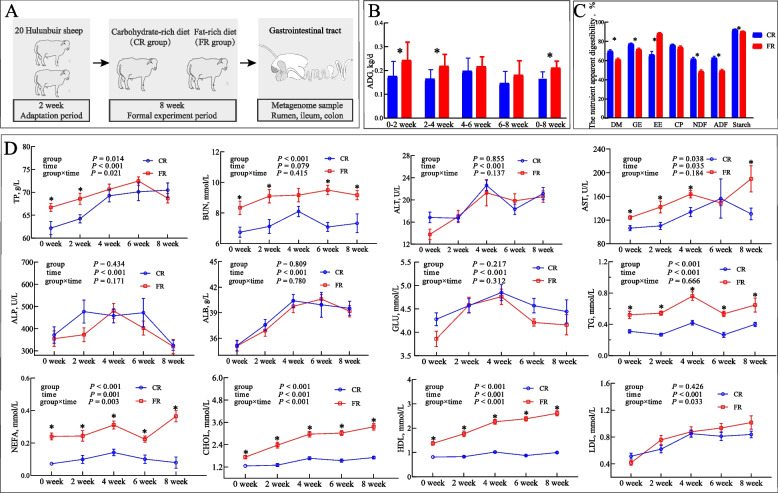
The fat-rich diet induced weight gain and altered lipid metabolism of the host sheep. **A** Schematic overview of the study design and sample collection. **B** The average daily gain (ADG) was monitored every 2 weeks within the dietary intervention. **C** Comparison of the apparent digestibility of sheep fed the carbohydrate-rich (CR group) and fat-rich diet (FR group). **D** Variation of serum biochemical profile of sheep fed a fat-rich diet for 8 consecutive weeks. ^*^P < 0.05. TP, total protein; BUN, blood urea nitrogen; ALT, alanine aminotransferase; AST, aspartate aminotransferase; ALP, alkaline phosphatase; ALB, albumin; GLU, glucose; TG, triglycerides; NEFA, non-esterified fatty acid; CHOL, cholesterol; HDL, high-density cholesterol; LDL, low-density cholesterol

Sheep were adapted to a fat-rich diet by gradually increasing the proportion of dietary fat from 5% to 24.4% over the adaptation period (Fig. 1A). Throughout the entire experimental period, the sheep maintained a good condition with normal feed intake and feces. Compared with the CR group, the ADG of sheep in the FR group was significantly increased (CR: 0.17 kg/d vs. FR: 0.21 kg/d, *P* = 0.003), especially during 0–2 weeks (*P* = 0.047) and 2–4 weeks (*P* = 0.019). No difference (*P* > 0.05) was observed during 4–6 weeks and 6–8 weeks (Fig. 1B), indicating an increased growth at an earlier stage.

The fat-rich diet did not alter the apparent digestibility of CP (*P* = 0.125), but decreased that of DM (*P* < 0.01), GE (*P* = 0.001), NDF (*P* < 0.01) and ADF (*P* < 0.01). The apparent digestibility of starch (*P* = 0.052) weakly decreased in the FR group (Fig. 1C). Conversely, the FR diet increased the apparent digestibility of EE (*P* < 0.01).

The high-fat diet significantly enhanced the concentrations of TP (*P* = 0.014), BUN (*P* = 0.038), and AST (*P* = 0.038) at various time points, but did not change ALT, ALP, GLU, and LDL (*P* > 0.05). In terms of lipid metabolism, the FR diet significantly enhanced the concentrations of TG (*P* < 0.001), NEFA (*P* < 0.001), CHOL (*P* < 0.001), and HDL (*P* < 0.001) at all time points (Fig. 1D).

### The fat-rich diet suppressed fiber-degrading bacteria and enhanced fat-utilization microbiota in region-specific patterns

**Fig. 2:**
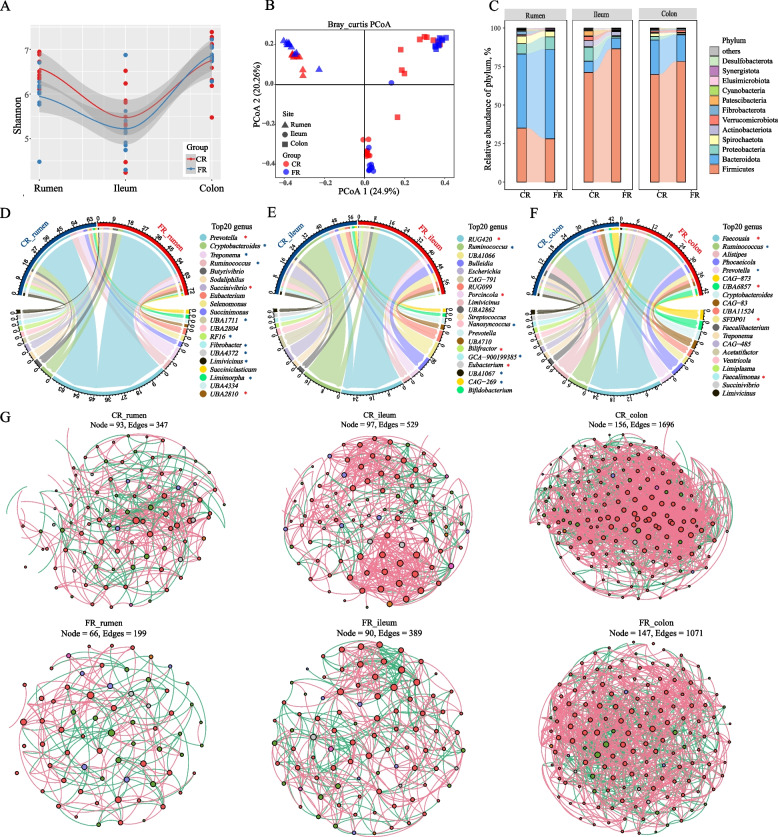
Different energy sources shape distinct rumen microbiota composition of sheep fed the carbohydrate-rich (CR group) and fat-rich diet (FR group). **A** Alpha diversity (Shannon). **B** Principal coordinate analysis (PCoA) among three gastrointestinal regions (rumen, ileum, colon) community structure based on Bray-Curtis dissimilarity. **C** Relative abundance of dominant phyla between two groups (average relative abundance > 0.1 %). **D–F **Changes in the relative abundance of Top20 genera between two groups in rumen (**D**), ileum (**E**), and colon (**F**). ^*^P < 0.05, blue: higher in CR group, red: higher in FR group. **G** Co-occurrence network at the genus level among three gastrointestinal regions (rumen, ileum and colon) of sheep fed the carbohydrate-rich (CR group) and fat-rich diet (FR group). Nodes represent a genus (relative abundance > 0.1%), and only significant (Pearson’s correlation, |*r*| > 0.5, *P* < 0.05) relationships are shown in solid lines. The red line indicates a positive correlation, while the green line shows a negative correlation

The FR diet elevated the Shannon index of alpha diversity in the rumen (*P* < 0.01), and reduced that in colon (*P* = 0.023, Fig. 2A). In addition, the GIT samples were partitioned into three distinct clusters, corresponding to different physiological areas (Fig. 2B). Principal coordinate analysis (PCoA) revealed that gastrointestinal region exerted a more pronounced effect on the separation of microbial composition than diet, but both have significant effect on microbial composition (PERMANOVA, *R*^2^_region_ = 0.425, *R*^2^_diet_ = 0.063, *R*^2^_region×diet_ = 0.075, F_region_ = 25.79, F_diet_ = 7.67, F_region×diet_ = 4.58, *P* = 0.001). There was a much clearer separation in the taxonomy classification of rumen and ileum under the dietary fat effect (*R*^2^_rumen_ = 0.253, *R*^2^_ileum_ = 0.305, *R*^2^_colon_ = 0.156, *P* = 0.001, Fig. S2).

The GIT microbiome was mostly assigned to bacterial taxa, and the abundance of bacteria in the rumen (*P* = 0.003) and ileum (*P* = 0.003) was significantly increased between the dietary treatment groups (Fig. S3). The bacteria across the gastrointestinal tract were dominated by Bacteroidota (61.39%), Firmicutes (26.94%), and Proteobacteria (4.59%). Among the dominant phyla, Firmicutes were elevated in the ileum and colon, and Bacteroidota were increased in rumen of FR group. The relative abundance of Verrucomicrobiota and Fibrobacterota decreased in both rumen and ileum of the FR group (Fig. 2C, Table S3). Within the top 20 genera, *Prevotella* (1.61-fold higher, *P* = 0.009), *Succinivibrio* (2.82-fold higher, *P* = 0.017), and *UBA2810* (*P* = 0.035) were enriched in the rumen of the FR group compared with the CR group. In contrast, *Cryptobacteroides* (1.43-fold lower, *P* = 0.027), *Treponema* (1.39-fold lower, *P* = 0.035), *Ruminococcus* (2.10-fold lower, *P* = 0.019), *Fibrobacte*r (10.5-fold lower, *P* < 0.01), *UBA1711* (*P* < 0.01), *RF16* (*P* = 0.001), *UBA4372* (*P* = 0.005), *Limivicinus* (*P* = 0.006), and *Limimorpha* (*P* = 0.044) were reduced in rumen of FR group (Fig. 2D, Table S4). For ileal microbiota, the relative abundance of the *RUG420* genus (family_Acutalibacteraceae) in the FR group was six-fold higher (FR27.36% vs. CR4.63%, *P* < 0.01) than in the CR group (Fig. 2E). In addition, *Porcincola* (*P* = 0.043), *Bilifractor* (*P* = 0.017) and *Eubacterium* (*P* < 0.01) were also increased in the FR group (Fig. 2E, Table S4). Conversely, the proportion of *Ruminococcus* (*P* = 0.001),* Nanosyncoccus* (*P* < 0.01), *GCA-900199385* (*P* = 0.022), *UBA1067* (*P* = 0.001), and *CAG-269* (*P* = 0.009) was reduced in the FR group compared with the CR group (Fig. 2E, Table S4). For colon, genus *Faecousia* (CR11.06% vs. FR16.91%, *P* = 0.035) and *Faecalimona*s (*P* < 0.01) were significantly enriched in the FR diet group, whereas *Ruminococcus* (CR6.1% vs. FR1.5%, *P* = 0.019), and *Prevotella* (CR3.5% vs. FR1.1%, *P* = 0.013) were decreased in the FR group (Fig. 2F). Interestingly, the *Fibrobacter*, *RF16*, *Limivicinus* in rumen were inversely correlated with ADG and lipid metabolic phenotypes of the host (Fig. S1). The *RUG420*, *Eubacterium* and *Porcincola* in the ileum and *Faecalimonas*, *SEDP01*, and *Faecousia* in the colon were positively correlated with these phenotypes.

To determine potential microbial interactions, six bacterial co-occurrence networks (|*r*| > 0.5, *P* < 0.05) were constructed for the genera with more than 0.1% abundance. The complexity of the network increased strongly along the gastrointestinal region, the microbial network node and connectivity in colon bacteria were the highest (Fig. 2G). Regarding the effect of diet, the CR-specific network was more complex than that of the FR group throughout the gastrointestinal bacteria as indicated by increased node and edge number, degree, and density (Table S5).

### The fat-rich diet led changes in the carbohydrate metabolism of rumen and colon

**Fig. 3:**
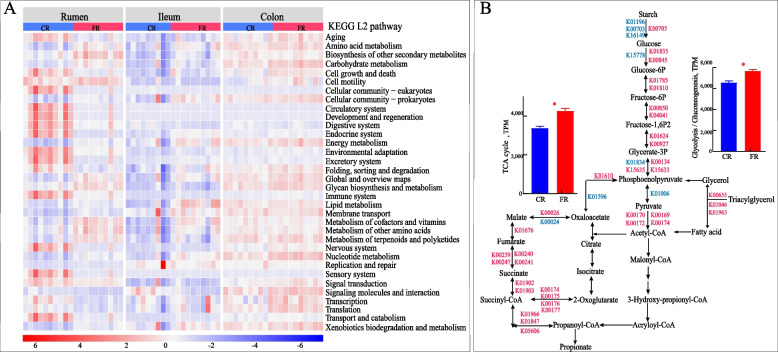
Changes in microbial function. **A** Heat map showing the KEGG pathways of three gastrointestinal regions at KEGG level 2 between the carbohydrate-rich (CR group) and fat-rich diet (FR group). **B** Significant KO involved in carbohydrate metabolism of rumen microbiome. ^*^P < 0.05, blue: higher in CR group, red: higher in FR group. Red text indicates the significantly increased KO in the FR group, while blue text indicates the significantly decreased KO in the FR group

KEGG pathway analysis were conducted to assess the metabolic capacity of the GIT microbiome. PCoA results showed that the gastrointestinal region and diet treatment contributed to the difference at KO level (PERMANOVA, F_region_ = 40.57, F_diet_ = 11.02, F_region_ × F_diet_ interaction = 4.97, *P* = 0.001), with a much clearer separation in the rumen and ileum (Fig. S4), suggesting that the GIT microbiome exhibits substantial functional heterogeneity. A total of 32, 21, and 7 KEGG L2 pathways were enriched in the rumen, ileum, and colon of the FR group, respectively (Fig. 3A, Fig. S5, Table S6). Based on the differences in dietary energy structure between carbohydrate and lipid, we observed the enrichment of carbohydrate metabolism and energy metabolism shared across the three regions, and the enrichment of lipid metabolism in the rumen and ileum of FR group (Table S6).

A total of 108 pathways showed increased relative abundances at KEGG level 3 in the rumen of the FR group (Table S7). These included the pathways for glycolysis/gluconeogenesis, citrate cycle, oxidative phosphorylation, propanoate metabolism, and pyruvate metabolism (*P* < 0.01) within the category of carbohydrate and energy metabolism. The FR diet enriched the abundance of genes related to propionate production (K01847, K01966, and K05606). Additionally, most differential genes in the TCA cycle were up-regulated in the FR group (Table S8). However, the KO linked to the catalytic conversion of starch to glucose (K00703, K01196, and K16149) was decreased in the rumen, suggesting a reduced carbon source and precursor from dietary carbohydrates. Given the enhanced abundance of genes (K0161, K04041) related to the irreversible reaction in gluconeogenesis, we further focused on the pathway involving non-sugar compounds such as glycerol and glycogenic amino acids. The enrichment of lipolytic enzymes (K00655, K01046, and K01963) in the rumen microbiome supported the degradation of triacylglycerol to glycerol, which then participates in gluconeogenesis. Moreover, the pathways of alanine metabolism, valine, leucine, and isoleucine degradation, phenylalanine metabolism, arginine and proline metabolism, histidine metabolism, cysteine and methionine metabolism, glycine, serine and threonine metabolism, and alanine, aspartate and glutamate metabolism were specifically up-regulated in the rumen of FR group (*P* < 0.01, Table S7). Lysine biosynthesis, Valine, leucine and isoleucine biosynthesis, and Phenylalanine, tyrosine and tryptophan biosynthesis were also enriched in the FR group (*P* < 0.01). The enrichment of genes (K01846, K04835, K19268, and K15024, Fig. S6) involved in glyoxylate and dicarboxylate metabolism and propanoate metabolism via the formation of propionyl-CoA was observed in the colon of the FR group.

### The fat-rich diet affected ileal lipid metabolism through bile acid modifications

**Fig. 4:**
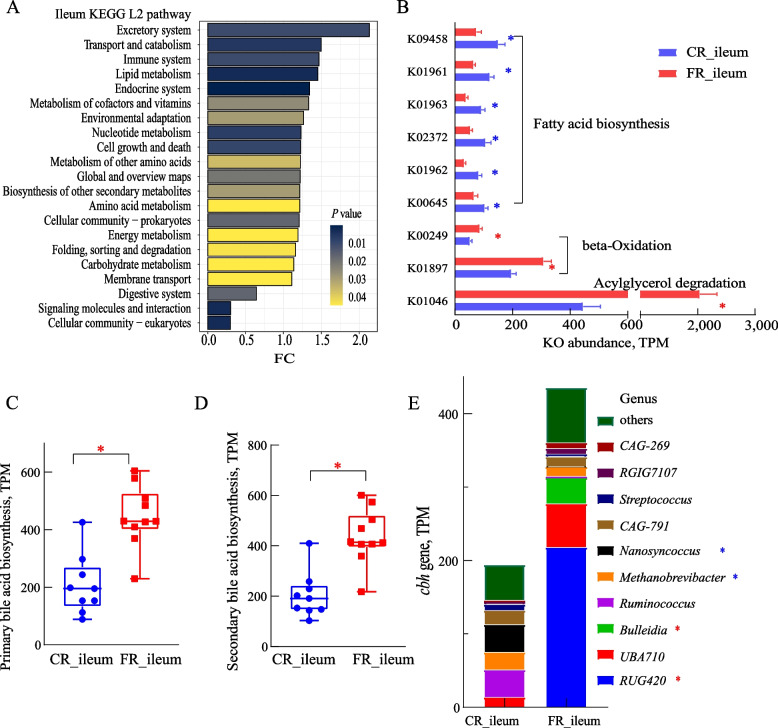
The lipid metabolism in the ileum is driven by altered gut microbiota mediated bile acid metabolism in sheep fed a fat-rich diet. **A** The significantly changed KEGG pathways of the ileum microbiome at KEGG level 2 between the carbohydrate-rich (CR group) and fat-rich diet (FR group). **B** Significant KO involved in lipid metabolism of the ileum microbiome. **C** and **D **The enrichment of primary bile acid biosynthesis and secondary bile acid biosynthesis. **E** Phylogenetic distribution of ileum microbial *cbh* (choloylglycine hydrolase) gene involved in bile acid deconjugation at the genus level. ^*^P < 0.05, red: higher in FR group, blue: higher in CR group

A total of 66 third-level pathways, involved in lipid, amino acid, energy, and carbohydrate metabolism of ileal microbiome, significantly changed between the two groups (Fig. 4A, Table S9). The fat-rich diet suppressed genes related to fatty acid biosynthesis in ileum microbiota, including K09458 (3-oxoacyl-acyl-carrier-protein synthase II, *P* = 0.023, 2.01-fold lower), K01961 (acetyl-CoA carboxylase, *P* = 0.012, 1.87-fold lower), K01963 (acetyl-CoA carboxylase carboxyl transferase subunit beta, *P* = 0.015, 2.38-fold lower), K02372 (3-hydroxyacyl-acyl-carrier-protein dehydratase, *P* = 0.044, 1.99-fold lower), K01962 (acetyl-CoA carboxylase carboxyl transferase subunit alpha, *P* = 0.012, 2.53-fold lower), K00645 (acyl-carrier-protein S-malonyltransferase, *P* = 0.044, 1.56-fold lower). Notably, the high-fat diet promoted signature genes associated with fatty acid degradation, including K00249 (acyl-CoA dehydrogenase, *P* = 0.018, 1.65-fold higher) and K01897 (long-chain acyl-CoA synthetase, *P* = 0.036, 1.58-fold higher) in beta-oxidation, K00655 (1-acyl-sn-glycerol-3-phosphate acyltransferase, *P* = 0.036, 1.48-fold higher) and K01046 (triacylglycerol lipase, *P* = 0.005, 4.58-fold higher) in glycerolipid metabolism (Fig. 4B). Moreover, the FR diet significantly enhanced the abundance of enzymes involved in primary (*P* = 0.007, Fig. 4C) and secondary (*P* = 0.008, Fig. 4D) bile acid biosynthesis. The abundance of K01442 (*cbh*, choloylglycine hydrolase), associated with bile salt hydrolysis, was more than twice in the FR group (*P* = 0.012) than that of the CR group. The *cbh* genes were phylogenetically assigned to the genus *RUG420*, followed by *UBA710* and *Bulleidia* (Fig. 4E). The shifts in *RUG420* (*P* < 0.01, increases) and *Nanosyncoccus* (*P* < 0.01, decrease) corresponded with changes in the ileal microbial composition.

### The fat-rich diet promotes energy productivity by improving the levels of SLP and ETP in distinct microbiomes among GIT regions

**Fig. 5:**
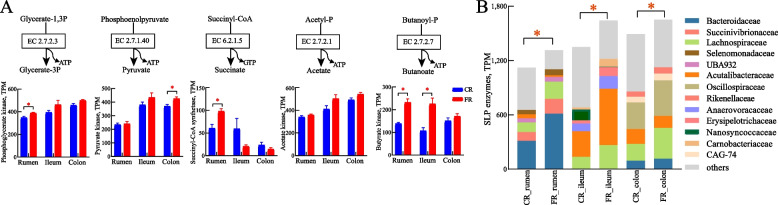
Major contributors to substrate-level phosphorylation (SLP) enzymes for ATP production among three gastrointestinal regions. **A** The five SLP reactions are coupled to the glycolysis, pyruvate metabolism, TCA cycle, and butanoate metabolism. **B** The relative abundance and taxonomic distribution of SLP enzymes at family level. ^*^P < 0.05, red: higher in fat-rich diet (FR) group

Notably, the significantly increased level of energy metabolism pathways was shared across three regions. For individual SLP enzymes, the abundance of phosphoglycerate kinase (*P* = 0.027), succinyl-CoA synthetase (*P* = 0.010), and butyrate kinase (*P* = 0.001) in the rumen, butyrate kinase (*P* = 0.005) in the ileum, and pyruvate kinase (*P* = 0.027, Fig. 5A) in the colon were higher in the FR group. The total abundance of SLP enzymes was significantly increased in rumen (*P* = 0.001), ileum (*P* = 0.001) and colon (*P* < 0.01, Fig. 5B). Moreover, Bacteroidaceae, Acutalibacteraceae, and Oscillospiraceae were the major contributors in rumen, ileum, and colon, respectively (Fig. 5B). Among the top 5 contributors in each region, significant increases were observed in Bacteroidaceae (*P* = 0.004) in the rumen and Lachnospiraceae (*P* = 0.011) in the colon, while Nanosyncoccaceae (*P* = 0.006) in the ileum significantly decreased when the diet shifted to FR.

**Fig. 6:**
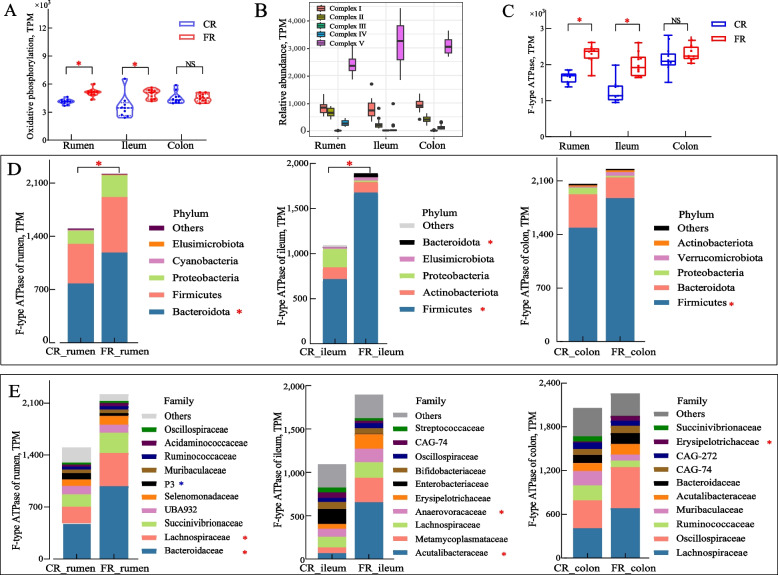
Comparisons of the abundance of oxidative phosphorylation components and major contributors to ATPase for ATP synthesis among three gastrointestinal. **A** and **B **The abundance of oxidative phosphorylation and their specific component among three gastrointestinal regions. **C** Comparison of the abundance of F-types ATPase between the two groups. **D** and **E** Variation in the abundance of F-types ATPase in the three gastrointestinal regions at phylum and family levels between the two groups. ^*^P < 0.05, red: higher in fat-rich diet (FR) group, blue: higher in carbohydrate-rich (CR) group

Total abundance of oxidative phosphorylation in the rumen (*P* < 0.001) and ileum (*P* = 0.006) of the FR group were higher than those of the CR group, with no observed difference in the colon (*P* = 0.684; Fig. 6A). Five membrane-associated complexes were involved in electron transport and ATP synthesis. The complexes I, II, IV, and V of the rumen microbiome and complexes I and V of the ileum microbiome showed increased abundance in the FR group (Fig. S7). As the final step of ETP, complex V, known as ATP-synthase, had the uppermost abundance in each distinct GIT (Fig. 6B). The ATPase is divided into three sub-families: F-type ATPase, V-type ATPase, and V/A-type ATPases (Fig. S8). The F-type ATPase showed the greatest variability with the dietary shift (Fig. S9). The expansion of F-ATPase genes was observed in the ileum of the FR group, followed by those in the rumen. Furthermore, the ileum of sheep in the FR group showed a 1.73-fold higher increase in F-type ATPase (FR1900 vs. CR1096, *P* < 0.001) compared to the CR group, followed by the rumen with a 1.47-fold increase (FR2238 vs. CR1523, *P* < 0.001); no difference was noted in the colon (Fig. 6C). The F-type ATPase biosynthetic genes were phylogenetically assigned to the phyla Firmicutes (63.54%), followed by Bacteroidetes (23.39%) and Proteobacteria (7.00%; Fig. 6D). Bacteroidetes was the predominant phylum assigned in the rumen, whereas Firmicutes played a crucial role in F-type ATPase biosynthesis in the intestine (ileum and colon). These phyla, with the highest abundance for F-type ATPase in all GIT regions, were significantly enriched in the FR group. The gene families were commonly classified into the Bacteroidaceae and Lachnospiraceae in the rumen, Acutalibacteraceae and Metamycoplasmataceae in the ileum, and Lachnospiraceae and Oscillospiraceae in the colon (Fig. 6E). Among them, there was an obvious increase in rumen contributors in the FR group, including Bacteroidaceae (2.03-fold higher, *P* = 0.002) and Lachnospiraceae (2.04-fold higher, *P* = 0.044). Acutalibacteraceae, the marker family in the ileum, was tenfold more abundant in sheep from the FR group (*P* = 0.006). Furthermore, the abundance of Anaerovoracaceae in the ileum and Erysipelotrichaceae in the colon were also significantly increased.

To better illustrate the connection between taxonomic shifts and functional changes, we conducted Pearson’s correlation analysis to determine the relationship between the SLP enzyme-related microbiome, F-type ATPase-related microbiome and host phenotype and microbial metabolic pathway (Fig. S10). The heatmap showed that the Bacteroidaceae, Lachnospiraceae and Succinivibrionaceae associated with ATP synthesis in rumen were positively associated with host ADG and microbial carbohydrate, lipid and energy metabolism. Acutalibacteraceae associated with ATP synthesis in ileum was positively associated with host ADG and microbial lipid metabolism. Oscillospiraceae associated with ATP synthesis in colon was positively associated with microbial carbohydrate metabolism. Further analysis, using multiple regression on matrices, revealed that the ATP-related microbiome encoding SLP and ETP enzyme in rumen, ileum, and colon contributed 36.95% to the host’s weight variation.

## Discussion

The nutritional status is related to systemic metabolic homeostasis [29, 30]. The sheep fed a fat-rich diet showed an increased in body weight despite a disturbed lipid profile, characterized by increased serum levels of TG, NEFA, CHOL, and HDL. This finding is consistent with previous observations in mice, pigs, and humans [31–33]. FR diet feeding worsened serum AST, suggesting the mitochondrial stress caused by fatty acid β-oxidation. Moreover, the significant weight gain primarily occurred during the first four weeks of the fat-rich diet stimulation. The rapid weight increase may be attributed to accumulation of TG and CHOL and expansion of adipose tissue response to acute caloric surplus, which varied dramatically in the first 4 weeks [34–36]. The subsequent slowdown is likely associated with increased thermogenesis and adaptive adjustments in energy homeostasis [37]. Carbohydrates and fat, as the primary energy-yielding macronutrients, are considered the main determinants in the transformation of energy fuels [38]. Generally, with sufficient carbohydrate intake, glucose is the optimal and preferred fuel for both the host and microbiota. When carbohydrate availability is reduced, fewer fluctuations in the circulating glucose and insulin levels create a pseudo-starvation metabolic state. This state induces a pronounced shift in metabolic fuel utilization, elevating lipid metabolism and ketone utilization as the primary energy source during carbohydrate deprivation [39, 40]. In our study, replacing dietary carbohydrates with fats led to marked increases in NEFA and TG, strongly indicating a heightened reliance on fat oxidation for fuel utilization and energy turnover. Consistent with the previous studies, our findings indicate that a fat-rich diet decreases nutrient digestibility, especially the NDF and GE, likely due to the sensitivity of fibrolytic bacteria and inhibition of enzyme activity [41, 42].

The gastrointestinal microbiome, as a key interface for energy capture, is able to convert dietary substrates into host nutrition in a composition-dependent manner [43, 44]. Accumulating evidence suggests a strong link between the commensal microbial community in the gastrointestinal tract and both animal growth potential and the risk of aberrant metabolism [45, 46]. Hence, understanding how diet and microbiota interact is a key issue in nutrition. We conducted an integrated analysis of microbial composition and functions (including lipid, carbohydrate, and energy metabolism) to clarify and compare the underlying mechanisms in the rumen, ileum, and colon affected by dietary fat intervention. The first finding was that the alpha diversity in the microbiomes of both the rumen and colon decreased. A possible explanation for this phenomenon could stem from the more specialized metabolic abilities of simpler species and metabolic networks [47]. Interestingly, the supreme disparity was observed in beta diversity in the ileum. Since digestion and absorption of fat mainly occur in the small intestine, modulating the microbial population and metabolic status of the ileum could create an adaptive microenvironment favorable to a fat-rich diet [48]. Among the taxonomic populations, the abundance of fiber-degrading microorganisms in the rumen, specifically those in the Fibrobacterota phylum and *Fibrobacter* and *Ruminococcus* genus, decreased. Given that the genus *Prevotella* is a major contributor to propionate production, insulin resistance, and immunoregulation [49, 50], its high abundance in the rumen is associated with the up-regulation of the propionate pathway. This up-regulation contributes to hyperlipoidemia in sheep fed a high-energy diet. The abundance of Firmicutes in the ileum was greater, potentially enabling more effective utilization of energy sources [7]. In addition, the enrichment of genera *RUG420* (family Acutalibacteraceae) and *Eubacterium* in ileum constituted a major reservoir of bile salt hydrolases, essential for bile acids and cholesterol transformations in lipid metabolism, consistent with the previous research [51, 52]. Minor changes were observed in the colon microbiome characterized by a decrease in the phylum Patescibacteria and an increase in the genera *Faecousia* and *Faecalimonas*. The dominant genera in the colon were *Faecousia* and *Ruminococcus*, indicating their main function of releasing energy from dietary carbohydrate that escape digestion by the host and foregut microbial enzymes [53, 54]. These results verify that differences in nutrition availability and the capacity to utilize substrates partially explain variations in the microbiota profile.

Variations in nutrient availability may lead to significant segmental differences in the microbial composition and function across different regions of the GIT [46]. These remarkable region-specific changes in microbial composition are likely to result in the segregation of their corresponding functions across distinct carbohydrate and lipid metabolic pathways. To be specific, the abundance of KO involved in the catalytic conversion of starch to glucose decreased in the rumen, thereby diminishing the available carbon source and precursor. However, de novo synthesis of glucose through gluconeogenesis can still occur from non-carbohydrate sources, including glycerol derived from triacylglycerol degradation and the carbon skeletons of some amino acids [55]. In this research, the enrichment of lipolytic enzyme (*TGL2*) and glucogenic amino acids metabolic pathway (such as alanine and phenylalanine) in the rumen microbiome supports the hypothesis that amino acids and lipids act together to provide precursors, compensating for carbohydrate deficiency in the rumen. Then, high activity levels of *pckA* (K01610, phosphoenolpyruvate carboxykinase) and *fbp3* (K04041, fructose-1,6-bisphosphatase III) in gluconeogenesis further support this mechanism. Dietary fatty acids may be largely shunted to the small intestine in ruminants [56]. Since ileum is unique in its lipid digestion and absorption functions, ileum microbes are more efficiently involved in regulating the absorption and metabolism of these substances, in which the gut microbiota plays an important role in the bio-transformation of bile acid metabolism [57, 58]. Our experiments demonstrate that a fat-rich diet strongly stimulates the pathways of both primary and secondary bile acid biosynthesis in the ileum. Importantly, the abundance of *cbh* gene, a rate-limiting enzyme in bacterial bile acids metabolism in the ileum, is enriched under high-fat conditions. This enzyme catalyzes the deconjugation of bile acids, aiding in the reduction of blood cholesterol levels [59]. Moreover, the high-fat diet significantly reshaped the composition of microbiota related to bile acids, which is consistent with ileal metagenomic changes that showed an increased abundance of *RUG420* and reduced *Nanosyncoccus*. These integrated results highlight that bile acids are a significant host factor in shaping the gut microbiome under a fat-rich diet. Our findings in the ileum—such as the enrichment of Eubacterium and bile acid transformation pathways—align with human studies linking these taxa and metabolic functions to lipid metabolism.

It is noteworthy that a significant increase in energy metabolism pathways was observed across three regions, indicative of heightened energy metabolic activity. Lipids or carbohydrates would share similar downstream energy utilization after catabolism into available energy currencies such as nicotinamide adenine dinucleotide (NADH), flavin adenine dinucleotide, reduced (FADH_2_), and ATP [60, 61]. However, the metabolic switch from glucose to fatty acid metabolism is a key factor in the variations of ATP yields [62]. In microbial metabolism, ATP production occurs via two major mechanisms: substrate-level phosphorylation and electron transport phosphorylation. Studies have determined that redox potential varies with diet and is the main factor leading to diet-induced changes in the abundance of this functional group, so redox potential is a key factor that can be used as a shaping driver of community assembly within anaerobic gut microbial communities [63]. The total abundance of SLP enzymes was significantly increased in three regions with the shift to a fat-rich diet. In ETP, electrons derived from energy sources are moved by the electron transport chain to O_2_ in aerobic respiration or to other terminal electron acceptors (nitrate, sulfate, and fumarate) in anaerobic respiration. Thus, the transmembrane electrochemical potential drives the synthesis of ATP [64]. The oxidative phosphorylation system, which is fundamental to key to energy production, is facilitated by five enzyme complexes located in the ETP. In the present study, we evaluated the roles of ETP in the energy productivity of the ruminal microbiome, focusing on F-type ATPase due to its rate-limiting action and high abundance. The activity of F-type ATPase in the FR diet increased by about 48% and 73% in rumen and ileum, respectively, while no significant change was observed in the colon. Considering that several pathways, including lysine biosynthesis, valine, leucine and isoleucine biosynthesis, and phenylalanine, tyrosine and tryptophan biosynthesis, were enriched in the rumen, we proposed that both enhanced SLP and ETP significantly supported metabolic fuel for amino acid synthesis. The increased activities of F-type ATPase in the ileum microbiome suggest that ETP, rather than the SLP enzyme, may contribute more to ATP production. The proposed mechanism is that increased lipid availability in the small intestine stimulates the formation of NADH and FADH_2_ through β-oxidation of fatty acids, thereby enhancing substrate supply for oxidative phosphorylation [65]. Furthermore, the small intestine maintains a high internal oxygen level, promoting a microbial community, dominated by bacteria that use respiration for energy production [66]. Interestingly, regional differences in the major contributors of F-type ATPase activity were also observed along the digestive tract, from rumen to the colon in sheep. Most ATP producers in the rumen were assigned to members of the family Bacteroidaceae. In contrast, the higher proportions of the family Acutalibacteraceae and Erysipelotrichaceae, known for their bile salt hydrolysis capability, in the FR group suggest that these organisms can use respiratory electron acceptors to generate ATP and fuel microbial growth. This may reflect the adaptive characteristics of the ileum in response to environmental conditions and diet.

Further analysis, using multiple regression on matrices, estimated association between the ADG variation and ATP-related microbiome in different regions. This analysis revealed that the ATP-related microbiome encoding SLP and ETP in the rumen, ileum, and colon were associated with 36.95% of the host’s ADG variation. The effect of ATP-related microbiome in ileum is the largest, followed by colon, and rumen. These associations further decipher a potential role of microbiome-dependent ATP production in the host performance, thereby provides insights into strategies for altering the gastrointestinal ecosystem to maximize energy efficiency.

## Conclusion

Collectively, the sheep fed a fat-rich diet had a greater ADG, and increased the reliance on fat oxidation for fuel utilization. The gastrointestinal microbiome deal with fat-rich diet through the loss of ruminal fiber-degrading bacteria (genus_*Fibrobacter*) and enrichment of ileal microbiota for bile acid and lipid metabolism (genera *RUG420* and *Eubacterium*). Moreover, microbiota exhibited a predilection for carbohydrate and lipid metabolism, leading to differences in downstream ATP production. The increased substrate supply from β-oxidation of fatty acids to oxidative phosphorylation enhanced ATPase expression and corresponding microbial growth in the ileum. The fat-rich diet increased the ATP-producing capacity through electron transport phosphorylation by Bacteroidaceae in rumen and Acutalibacteraceae in ileum. Altogether, microbiome-dependent ATP synthesis in ileum made greater association to the host’s ADG under a fat-rich diet. These findings demonstrate the microbial potential in the ATP synthesis under the shift in dietary energy source, help better understanding how the gastrointestinal microbiota affect the growth performance of ruminants, and provide a new perspective on the energy metabolism and precise macronutrients nutrition.

## Supplementary Information

Additional file 1: Table S1 Ingredient and nutrient composition of the experimental diets. Table S2 Sequencing data of the 59 GIT samples among the three regions of lambs. Table S3 Regional differences in microbial taxa at the phylum level among three GIT regions in sheep fed carbohydrate-rich diet or far-rich diet. Table S4 Regional differences in microbial taxa at the genus level among three GIT regions in sheep fed carbohydrate-rich diet or far-rich diet. Table S5 Key features of Co-occurrence network at genus level (> 0.1%) among three gastrointestinal regions of sheep fed the carbohydrate-rich (CR group) and fat-rich diet (FR group). Table S6 Regional differences in KEGG pathway level 2 among the three GIT regions in sheep fed carbohydrate-rich diet or far-rich diet. Table S7 Differences in rumen microbiome metabolic pathways at KEGG level 3 in sheep fed carbohydrate-rich diet or far-rich diet. Table S8 The comparison of microbial genes related to rumen carbonhydrate metabolism of sheep fed carbohydrate-rich diet or far-rich diet. Table S9 Differences in ileal microbiome pathways at KEGG level 3 in sheep fed carbohydrate-rich diet or far-rich diet.

Additional file 2: Fig. S1 Pearson’s correlation matrix of significantly changed genera in three regions and ADG and lipid metabolic phenotypes. Fig. S2 Principal coordinate analysis (PCoA) of taxonomic community composition in the rumen (A), ileum (B), and colon (C) between two groups based on Bray-Curtis dissimilarity. Fig. S3 Comparison of microbial domains in rumen (A), ileum (B), and colon (C) of sheep fed the carbohydrate-rich (CR group) and fat-rich diet (FR group). Fig. S4 Principal coordinate analysis (PCoA) of KO genes in the rumen (A), ileum (B), and colon (C) between the two groups based on Bray-Curtis dissimilarity. Fig. S5 Heatmap showing the significantly changed pathways of the rumen (A), ileum (B), and colon (C) microbiome at KEGG level 2 between the two groups. Fig. S6 Significant KO involved in propanoate metabolism of colon microbiome. Fig. S7 The membrane-associated complexes (complexes I, II, III, IV and V) involved in electron transport and ATP synthesis across three gastrointestinal regions. Fig. S8 The abundance of three sub-families of ATPase across three gastrointestinal regions: the F-type ATPase, V-type ATPase and V/A-type ATPases. Fig. S9 Comparison of the abundance of ATPase between the two groups in rumen (A), ileum (B), and colon (C). Fig. S10 Correlation analysis of SLP enzyme-related microbiome, F-type ATPase-related microbiome and host phenotype and microbial metabolic pathway in rumen (A), ileum (B), and colon (C).

## Data Availability

Raw reads of metagenomic sequencing of rumen, ileum, and colon content are available at NCBI SRA (project number PRJNA972992).

## Abbreviations

ADF: Acid detergent fiber
ADG: Average daily gain
ADP: Adenosine diphosphate
ALB: Albumin
ALP: Alkaline phosphatase
ALT: Alanine aminotransferase
AST: Aspartate aminotransferase
ATP: Adenosine triphosphate
BUN: Blood urea nitrogen
BW: Body weight
CHOL: Cholesterol
CP: Crude protein
CR: Carbohydrate-rich diet
DM: Dry matter
DMI: Dry matter intake
EE: Ether extract
ETP: Electron transport phosphorylation
FADH_2_: Flavin adenine dinucleotide, reduced
FDR: False discovery rate
FR: Fat-rich diet
GIT: Gastrointestinal tract
GLU: Glucose
HDL: High-density lipoprotein cholesterol
LDL: Low-density lipoprotein cholesterol
NADH: Nicotinamide adenine dinucleotide
NDF: Neutral detergent fiber
NEFA: Non-esterified fatty acids
ORFs: Open reading frames
SLP: Substrate-level phosphorylation
TG: Triglyceride
TP: Total protein
TPM: Transcripts per million
