# Supplementary material for: Fat-rich diet promotes microbiome-dependent ATP synthesis in sheep model
Source: J Anim Sci Biotechnol. 2025 Jun 5;16:81. doi: 10.1186/s40104-025-01214-9 (PMC12139142; doi:10.1186/s40104-025-01214-9)
Supplement: Supplementary file 2 — Additional file 2: Fig. S1 Pearson’s correlation matrix of significantly changed genera in three regions and ADG and lipid metabolic phenotypes. Fig. S2 Principal coordinate analysis (PCoA) of taxonomic community composition in the rumen (A), ileum (B), and colon (C) between two groups based on Bray-Curtis dissimilarity. Fig. S3 Comparison of microbial domains in rumen (A), ileum (B), and colon (C) of sheep fed the carbohydrate-rich (CR group) and fat-rich diet (FR group). Fig. S4 Principal coordinate analysis (PCoA) of KO genes in the rumen (A), ileum (B), and colon (C) between the two groups based on Bray-Curtis dissimilarity. Fig. S5 Heatmap showing the significantly changed pathways of the rumen (A), ileum (B), and colon (C) microbiome at KEGG level 2 between the two groups. Fig. S6 Significant KO involved in propanoate metabolism of colon microbiome. Fig. S7 The membrane-associated complexes (complexes I, II, III, IV and V) involved in electron transport and ATP synthesis across three gastrointestinal regions. Fig. S8 The abundance of three sub-families of ATPase across three gastrointestinal regions: the F-type ATPase, V-type ATPase and V/A-type ATPases. Fig. S9 Comparison of the abundance of ATPase between the two groups in rumen (A), ileum (B), and colon (C). Fig. S10 Correlation analysis of SLP enzyme-related microbiome, F-type ATPase-related microbiome and host phenotype and microbial metabolic pathway in rumen (A), ileum (B), and colon (C). [file 40104_2025_1214_MOESM2_ESM.docx]

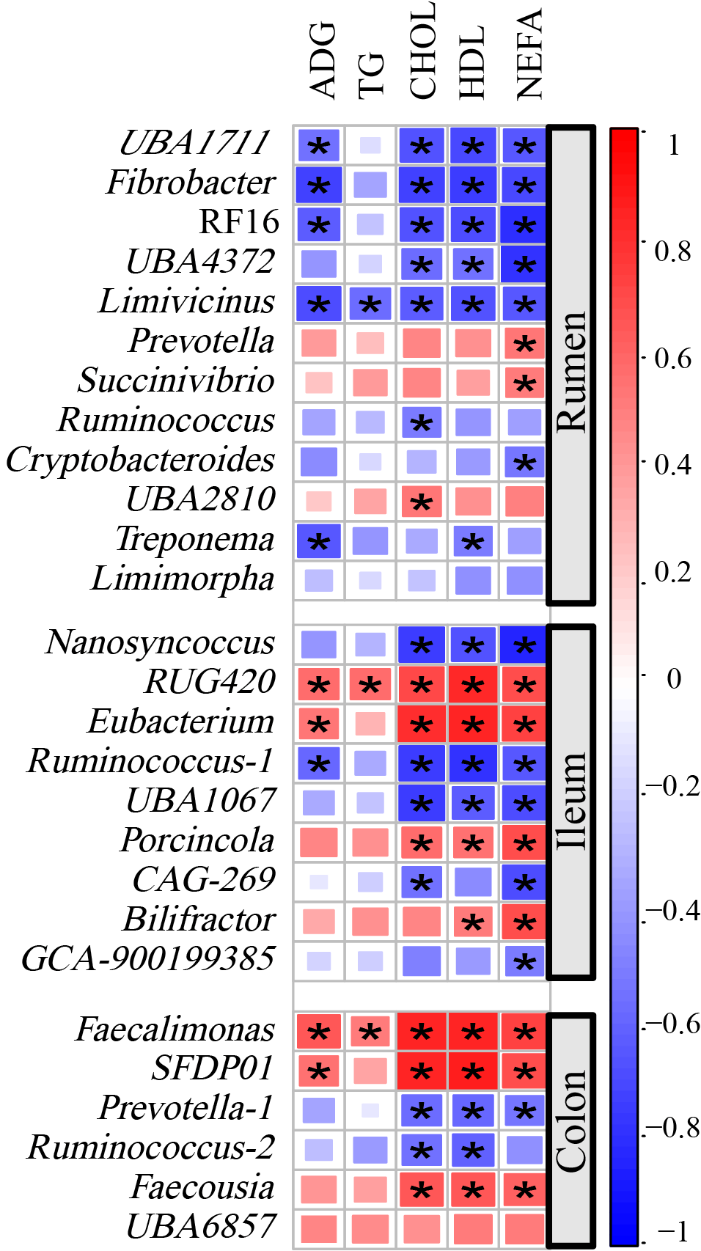


**Fig. S1** Pearson’s correlation matrix of significantly changed genera in three regions and ADG and lipid metabolic phenotypes. ^*^|*r*| > 0.5, *P* < 0.05. Red indicates positive correlation, and blue indicates negative correlation


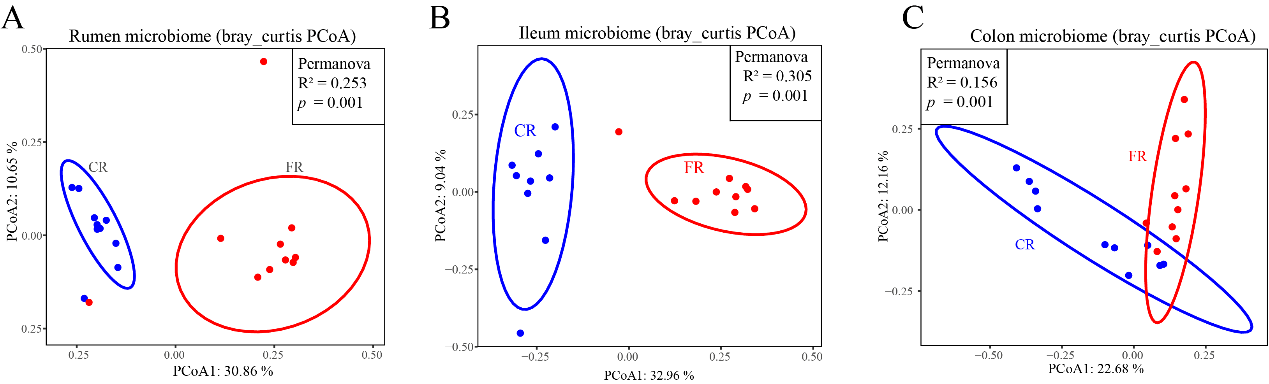


**Fig. S2** Principal coordinate analysis (PCoA) of taxonomic community composition in the rumen (**A**), ileum (**B**), and colon (**C**) between two groups based on Bray-Curtis dissimilarity


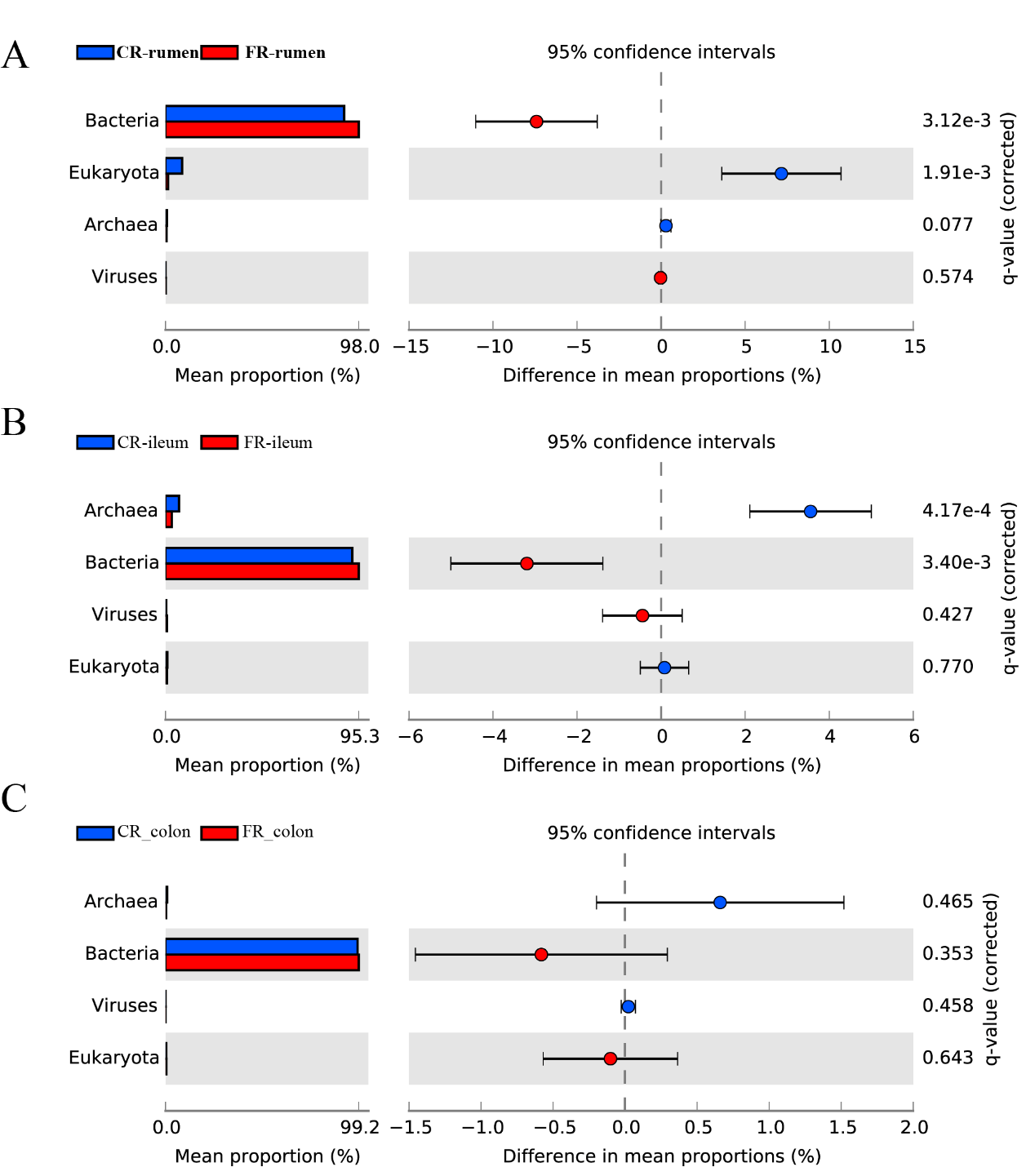


**Fig. S3** Comparison of microbial domains in rumen (**A**), ileum (**B**), and colon (**C**) of sheep fed the carbohydrate-rich (CR group) and fat-rich diet (FR group). Significantly different domains were tested by Welch's *t*-test with adjusted *P* <0.05


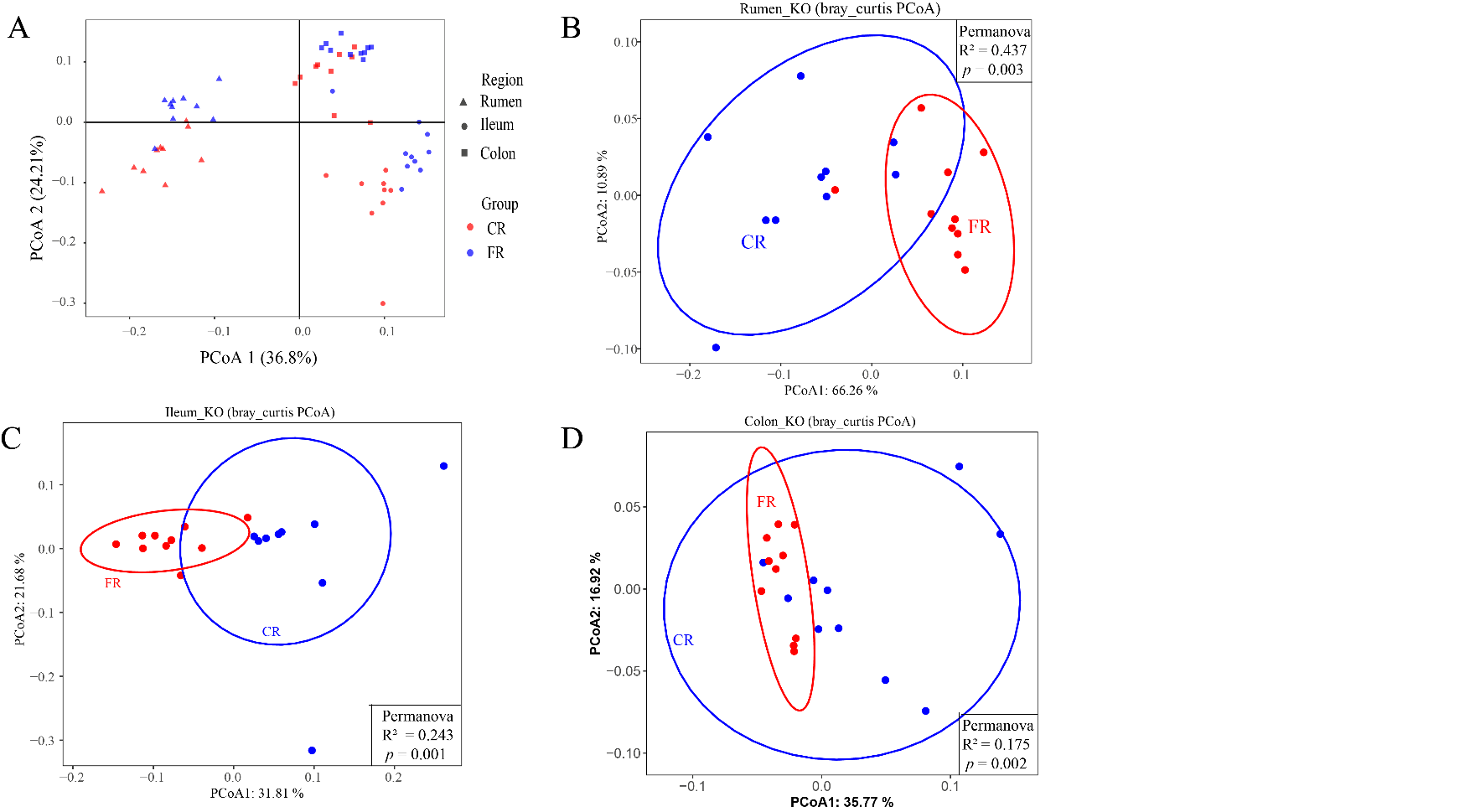


**Fig. S4** Principal coordinate analysis (PCoA) of KO genes in the rumen (**A**), ileum (**B**), and colon (**C**) between the two groups based on Bray-Curtis dissimilarity. CR: carbohydrate-rich diet, FR: fat-rich diet


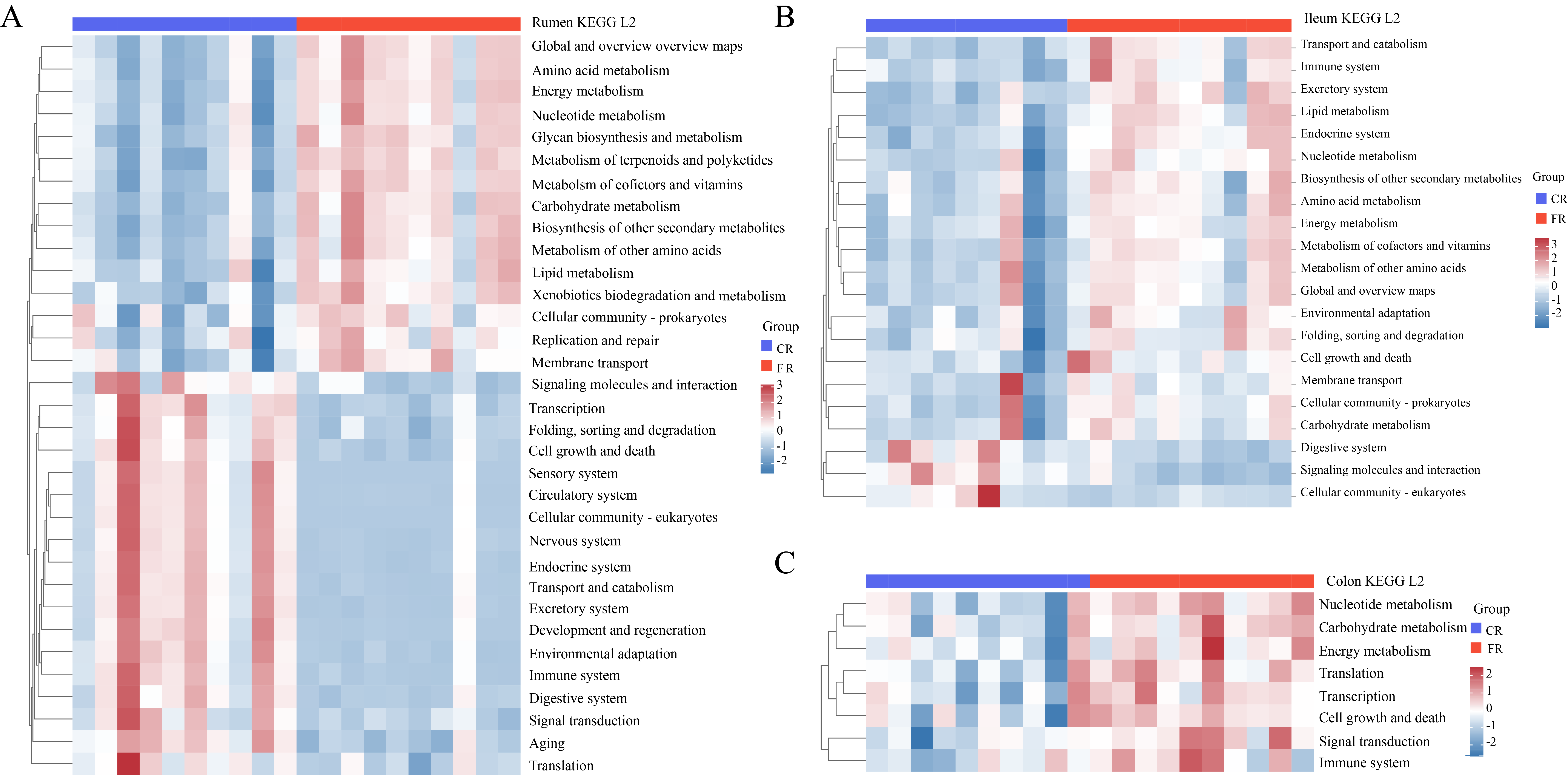


**Fig. S5** Heatmap showing the significantly changed pathways of the rumen (**A**), ileum (**B**), and colon (**C**) microbiome at KEGG level 2 between the two groups. CR: carbohydrate-rich diet, FR: fat-rich diet


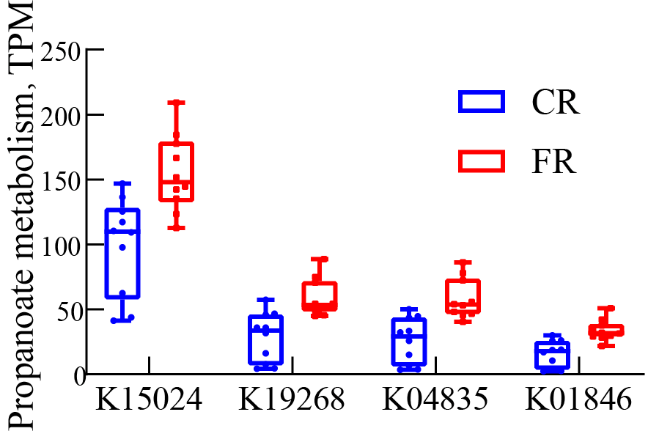


**Fig. S6** Significant KO involved in propanoate metabolism of colon microbiome. CR: carbohydrate-rich diet, FR: fat-rich diet


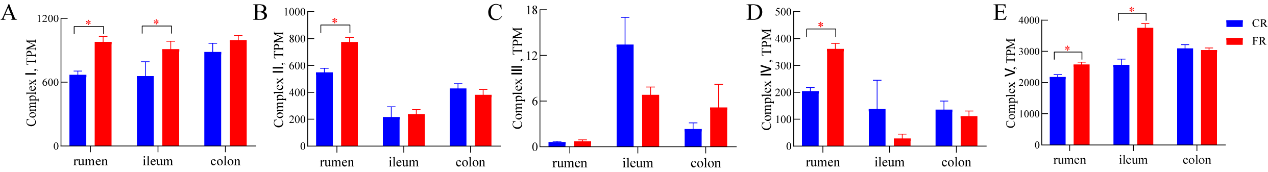


**Fig. S7** The membrane-associated complexes (complexes Ⅰ, II, III, Ⅳ and Ⅴ) involved in electron transport and ATP synthesis across three gastrointestinal regions. **A** Complexes Ⅰ: NADH dehydrogenase, **B** Complexes II: succinate dehydrogenase, **C** Complexes III: cytochrome c oxidoreductase, **D** Complexes Ⅳ: cytochrome c oxidase, and **E** Complexes Ⅴ: ATP synthase


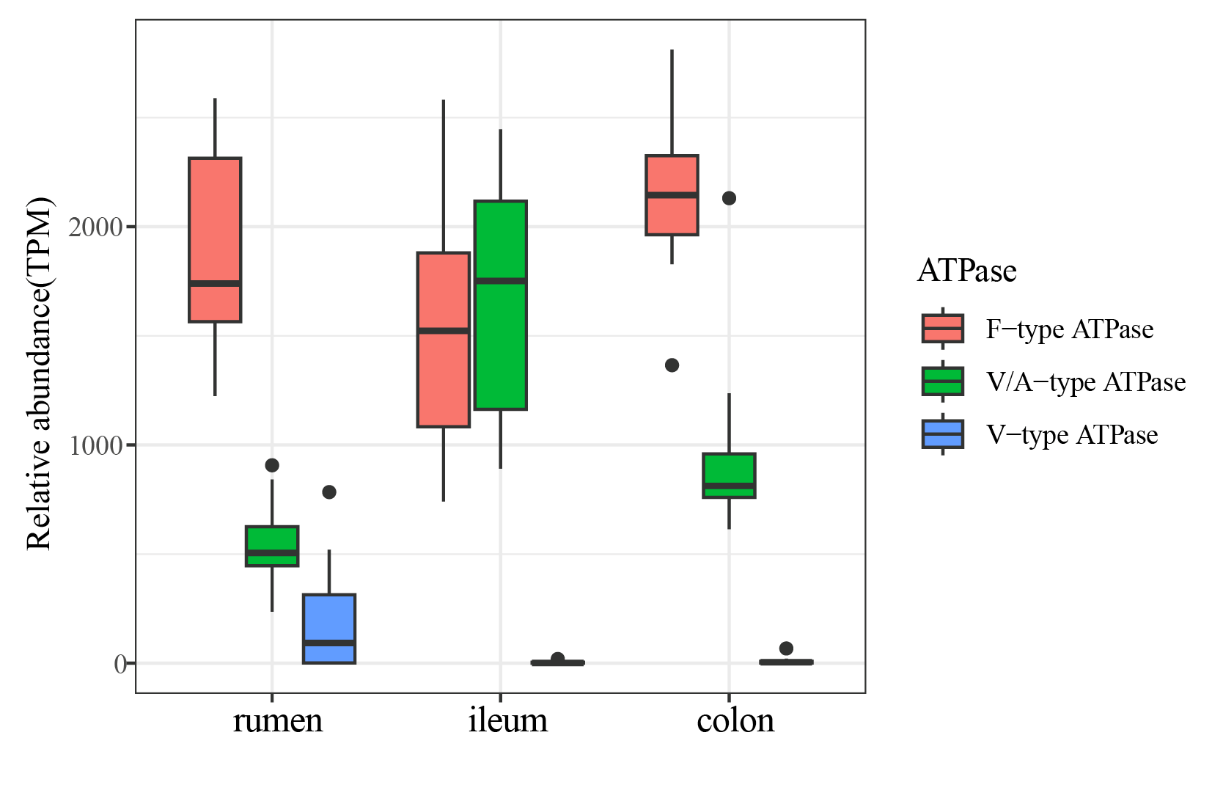


**Fig. S8** The abundance of three sub-families of ATPase across three gastrointestinal regions: the F-type ATPase, V-type ATPase and V/A-type ATPases


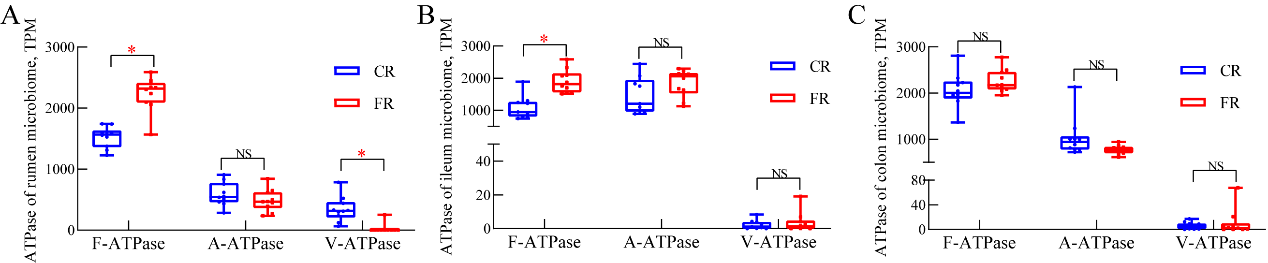


**Fig. S9** Comparison of the abundance of ATPase between the two groups in rumen (**A**), ileum (**B**), and colon (**C**). CR: the lambs fed carbohydrate-rich diet, FR: the lambs fed fat-rich diet


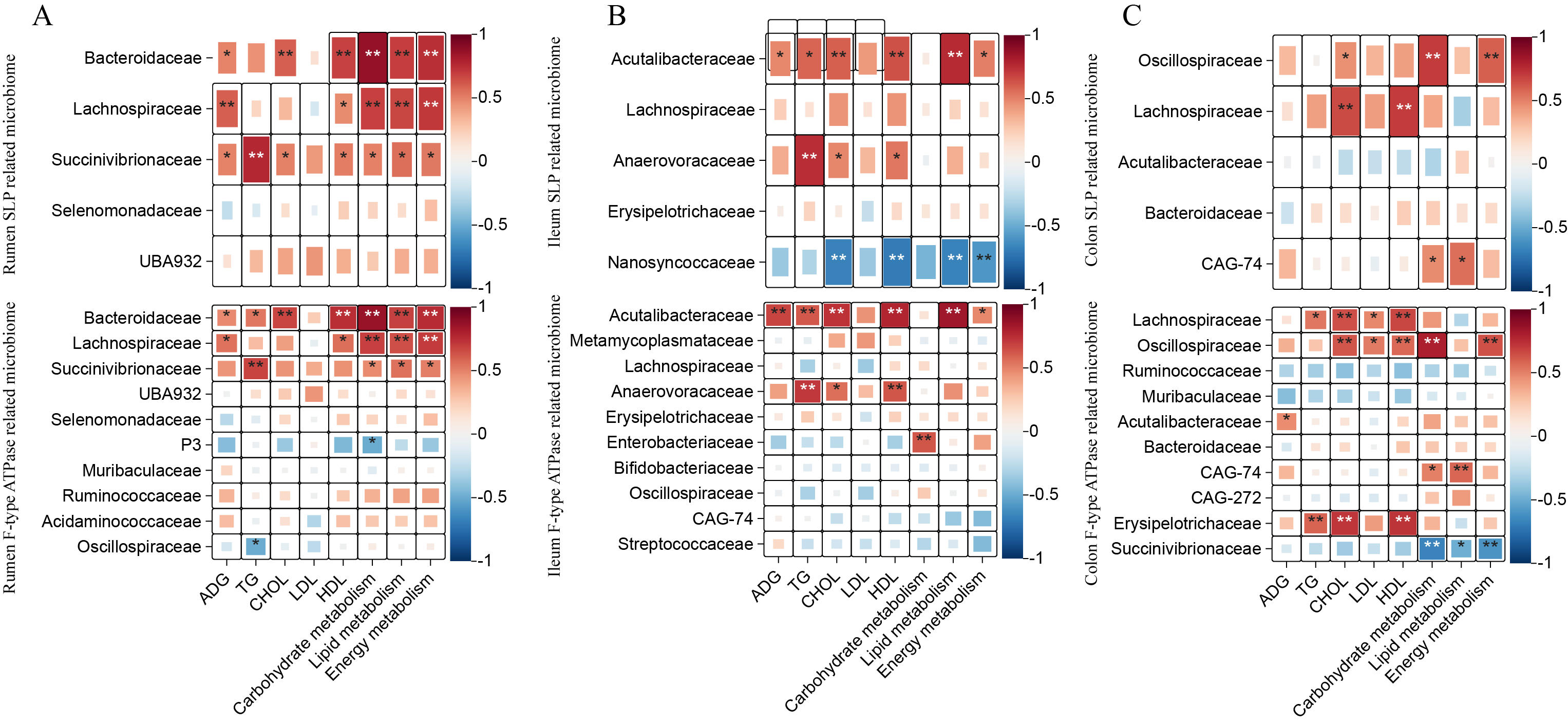


**Fig. S10** Correlation analysis of SLP enzyme-related microbiome, F-type ATPase-related microbiome and host phenotype and microbial metabolic pathway in rumen (**A**), ileum (**B**), and colon (**C**). ^*^|*r*| > 0.5, 0.01 < *P* < 0.05. ^*^|*r*| > 0.5, *P* < 0.01, Red indicates positive correlation, and blue indicates negative correlation
